# Hybrid Dual-Link Data Transmission Based on Internet of Vessels

**DOI:** 10.3390/s25061899

**Published:** 2025-03-18

**Authors:** Fei Li, Ying Guo, Ziqi Wang, Yuhang Chen, Jingyun Gu

**Affiliations:** College of Information Science and Technology, Qingdao University of Science and Technology, Qingdao 266061, China; lifei@mails.qust.edu.cn (F.L.); wangziqi@mails.qust.edu.cn (Z.W.); chenyuhang@mails.qust.edu.cn (Y.C.); gujingyun@mails.qust.edu.cn (J.G.)

**Keywords:** Internet of Vessels, dual-link data transmission, spatial crowdsourcing task allocation, multi-hop

## Abstract

The transmission of marine data is an urgent global challenge. Due to the particularity of underwater environments, the efficiency and reliability of data transmission in underwater acoustic communication are severely restricted, especially in long-distance and large-scale data transmission situations. This study proposes a dual-link data transmission method based on the Internet of Vessels, utilizing the powerful communication capabilities and flexibility of ships as relay nodes for data transmission. By constructing both above-water and underwater dual-link collaborative transmission, the method effectively improves data transmission rates and stability. Additionally, a spatial crowdsourcing allocation algorithm based on Bayesian reputation selection is designed to assess the capability of ships to complete tasks, and an integrated scoring function is used to select the optimal relay ship, solving the problems of relay ship selection and transmission path selection in the data transmission process. Furthermore, this study introduces an incentive mechanism for data transmission based on the Internet of Vessels, which maximizes the stability of data transmission. Experimental results show that the dual-link data transmission method of the Internet of Vessels significantly improves the reliability and transmission speed of underwater communication, providing a novel and practical solution for long-distance, large-volume data transmission in maritime environments.

## 1. Introduction

Data transmission plays a crucial role in marine networks. Efficient and real-time data transmission enables the precise monitoring and management of the marine environment, resources, and facilities, supporting marine disaster warning, ecological protection, and sustainable resource development [1]. Data collection devices, such as sensors, buoys, and unmanned submersibles, continuously gather key data on marine temperature, salinity, water quality, meteorological conditions, and more. The transmission system ensures that this information is delivered in a timely and accurate manner to shore-based or cloud platforms for analysis and processing [2]. Stable data transmission technologies effectively support intelligent applications such as autonomous navigation and underwater robotics [3], enhancing the efficiency of marine management and emergency response.

Data transmission in the marine environment faces numerous technical challenges. Underwater communication is heavily affected by seawater absorption and scattering, resulting in significant signal attenuation, especially in deep-water areas where the communication range and data transfer rates are limited. Bandwidth constraints make large-scale data transmission and real-time monitoring difficult, while noise interference in the marine environment further degrades signal quality [4]. The energy consumption of devices is another major issue, particularly for unmanned underwater devices, where the limited energy restricts the operational time. Additionally, transmission delays impact real-time applications such as disaster warning and environmental monitoring. Although technologies like acoustic communication have been applied, the complexity of the marine environment makes overcoming issues such as signal attenuation and bandwidth limitations a pressing challenge. Many scholars are actively researching this issue, but an ideal solution has yet to be found.

**Figure 1:**
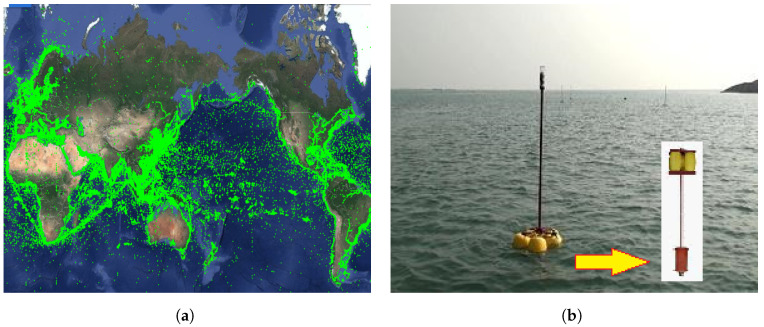
(**a**) Ship distribution. (**b**) Dual-head node.

In recent years, the development of marine Internet of Things (IoT) technology has been rapid, especially in utilizing surface vessels to form a vessel network for data transmission (as shown in Figure 1a), making it possible to leverage surface vessel resources to improve underwater communication performance. Compared to underwater networks, vessel networks offer several advantages, including faster transmission speeds, longer transmission distances, lower bit error rates, more stable links, lower energy consumption, easier deployment and maintenance, and a wider variety of devices. Against this backdrop, this paper proposes a Hybrid Dual-Link Transmission Algorithm (HDTA) based on the Internet of Vessels, aimed at improving underwater communication performance by utilizing vessel resources. Figure 1b shows the sensor nodes used in this study. These nodes were independently developed by the Sensor Network Laboratory in collaboration with our team and deployed on the OceanSense marine sensor network test platform in Qingdao Bay. These sensor nodes have bidirectional communication capabilities between above-water and underwater environments, allowing for effective data transmission across both, which we refer to as “dual-head nodes”. The OceanSense platform provides a real-world experimental environment for this study, used to validate the communication and data transmission capabilities of marine sensor nodes in complex oceanic conditions. In this study, we deploy bidirectional sensor nodes on vessels to verify their feasibility and performance in underwater and surface communications. These vessels can not only communicate with underwater sensor nodes but also exchange information with other vessels, thus establishing a dual-link communication model between the surface and underwater.

Specifically, the dual-link data transmission based on the vessel network faces two main challenges: first, how to select suitable vessels as relay nodes, and second, how to determine the data transmission path. To address these issues, this paper designs the HDTA algorithm transmission model and proposes a spatial crowdsourcing task allocation algorithm based on Bayesian reputation. By treating underwater nodes as task publishers and vessels as task executors, vessels participating in data transmission are selected through spatial crowdsourcing. The algorithm combines a comprehensive scoring function and a solution to the cold start problem to ensure the stability and efficiency of the data link.

The subsequent sections of this paper are organized as follows.

In Section 2, a review of the existing literature related to this study is provided, focusing on the main research progress and methods in the field. The strengths and weaknesses of various research methods are compared and analyzed. In Section 3, the research methodology is described, and a crowdsourcing allocation algorithm based on Bayesian reputation for selecting high-quality vessels is proposed. This section also addresses relay vessel selection and transmission path selection issues, combining a comprehensive scoring function and solutions to the cold start problem. Incentive schemes are introduced to ensure the stability of data transmission and encourage more vessels to participate. In Section 4, the research results are presented in detail through relevant experiments, followed by an in-depth discussion. These results are compared with the existing literature to demonstrate the feasibility and advantages of the proposed solution. Finally, in Section 5, a comprehensive summary of the research is provided, along with suggestions for future research directions.

## 2. Related Work

To improve the reliability of data collection in underwater environments, recent years have seen significant research into the use of Underwater Sensor Networks (UWSNs) for underwater data transmission. The findings in this area are highly relevant to this paper. Underwater Sensor Networks are an essential component of marine networks, wherein sensor nodes are deployed underwater, and communication links are established to enable the real-time monitoring, data collection, and information transmission of the marine environment [5,6]. However, unlike electromagnetic wave transmission on land, acoustic waves in water propagate more slowly and are influenced by phenomena such as multipath propagation, reflection, and diffraction. The path loss in underwater transmission is substantial, with severe signal attenuation and limited transmission distance [7]. Therefore, designing an effective mechanism that allows UWSNs to achieve long-distance, large-volume data collection and transmission under acoustic transmission conditions poses a significant challenge. Given the inherent limitations of UWSNs, current research methods in the literature can be categorized into two approaches: one is using multi-hop routing for data transmission, and the other is deploying flexible Autonomous Underwater Vehicles (AUVs) and Unmanned Underwater Vehicles (UUVs) to assist with data collection and transmission. In AUV-assisted transmission schemes, network models are generally divided into three categories [8]: (I) Distributed Networks [9,10,11]: nodes are independently distributed, and AUVs need to access each node, although this is less efficient. (II) Node grouping based on fixed AUV paths [12,13]: limited by the fixed AUV path and network architecture, the primary goal is to avoid conflicts between nodes and increase network throughput, but the fixed AUV path restricts the network’s flexibility. (III) AUV-assisted clustering networks [14]: network nodes are divided into clusters, and AUVs only collect data from cluster heads, significantly reducing the AUV travel time. By properly selecting cluster heads and carefully designing clustering algorithms, network performance can be significantly improved.

In multi-hop transmission methods, most UWSN data transmission approaches focus on energy consumption. Existing energy-efficient routing algorithms for UWSNs can be classified into three categories: depth-based routing algorithms [15,16], clustering-based routing algorithms [17,18,19], and cooperative reliability-based routing algorithms [20,21]. These algorithms aim to reduce energy consumption and delay node failure times. However, due to issues like network voids and the limitations of the algorithms themselves, even with sufficient live nodes in the UWSN, the network may still fail prematurely if an important node encounters problems and data cannot be transmitted effectively. To address the energy consumption of aggregation nodes in long-distance data transmission, methods using drone-assisted aggregation nodes for data transmission have been proposed [22]. Drone data collection offers higher flexibility, with lower deployment and transmission latency, and higher bandwidth, among other advantages, but requires reliable drone deployment or path planning algorithms [23]. To achieve collaborative communication and resource sharing in storage, a maritime data transmission scheme assisted by unmanned surface vehicles (USVs) has been proposed [24]. We summarize the comparison of the advantages and disadvantages of HDTA and other related references in certain aspects in Table 1.The checkmark indicates that the specific study in the reference demonstrates significant advantages in certain aspects compared to other studies.

However, most of the aforementioned studies still face significant challenges posed by the inherent limitations of underwater communications, such as severe path loss, limited transmission range, high signal attenuation, and problems related to multipath propagation and environmental interference. The number of vessels in the ocean has reached a certain scale. As relay nodes, vessels offer a larger communication coverage, stable energy supply, and high bandwidth support compared to AUVs, USVs, and buoys, making them suitable for long-distance, high-speed data transmission. Their strong maneuverability and flexibility enable them to adapt to dynamic environments, whereas AUVs and USVs are limited by underwater or surface constraints, and buoys are restricted by energy supply and fixed positions, resulting in a smaller communication range. Therefore, this research proposes using passing vessels as relay nodes in the multi-hop transmission method, employing a spatial crowdsourcing allocation algorithm to ensure the stability and rate of data transmission links. The concept of crowdsourcing was first introduced by Jeff Howe and Mark Robinson in 2006 [28]. With the development of mobile internet and the sharing economy, traditional crowdsourcing has shifted towards spatial crowdsourcing [29]. Task allocation is considered the most crucial issue in spatial crowdsourcing [30], and spatial crowdsourcing task allocation algorithms have been applied in various fields. Task allocation can be classified into two dimensions based on the arrival scenario: static matching and dynamic matching. Static matching involves the one-time matching of known task and worker information, whereas dynamic matching deals with real-time changing tasks and worker information, requiring real-time scheduling and response. Wu et al. [31] proposed a time-prediction-based task allocation framework based on historical data, and introduced a heuristic algorithm for the one-time assignment of spatial crowdsourcing tasks to appropriate workers. Dynamic matching addresses real-time variations, with algorithms designed for real-time adjustment. Yao et al. [32] addressed the problem of online non-rejection-aware task allocation in spatial crowdsourcing, and proposed a non-rejection threshold-based random algorithm (ONRTA-RT) to solve the issue. Zhao et al. [33] improved the allocation utility using reinforcement learning for dynamic online allocation.

Task allocation can also be categorized based on objectives, such as maximizing the number of tasks, minimizing total cost, or achieving stable matching and fair allocation. Zhao et al. [34] first employed Voronoi diagrams and adaptive weighted Voronoi algorithms for geographic partitioning, followed by reinforcement learning to maximize the total number of allocated tasks while minimizing the average task allocation differences. Zhao et al. [35] considered fairness in task allocation from the worker’s perspective and solved the Fair and Effective Task Allocation (FETA) problem. Huang et al. [36] proposed a combined solution with two suggested matching techniques to improve the overall matching utility and reduce the blocking ratio, considering worker preferences. Ma et al. [37] studied the multi-objective spatiotemporal task allocation (MOST) problem in spatial crowdsourcing, modeling it as a combinatorial multi-objective optimization (MOO) problem to maximize overall task completion rates and minimize average task time costs. Md Mujibur Rahman et al. [38] addressed the stable matching problem by using reputation to measure workers’ reliability, employing multi-criteria trust and reputation factors derived from a Mamdani fuzzy inference system to allocate each spatial task. In this study, we propose a Bayesian reputation-based spatial crowdsourcing allocation algorithm, which effectively assigns task information to the most suitable vessels, selecting the best vessels as relay nodes to ensure link stability.

## 3. Algorithm Design

### 3.1. HDTA Algorithm (Hybrid Dual-Link Transmission Algorithm Based on Internet of Vessels)

In the Internet of Vessels, ships are equipped with dual-head nodes, allowing them to communicate both with underwater nodes and with other ships on the surface. Due to the limited communication range, a multi-hop transmission method is employed for data transmission.

**Figure 2:**
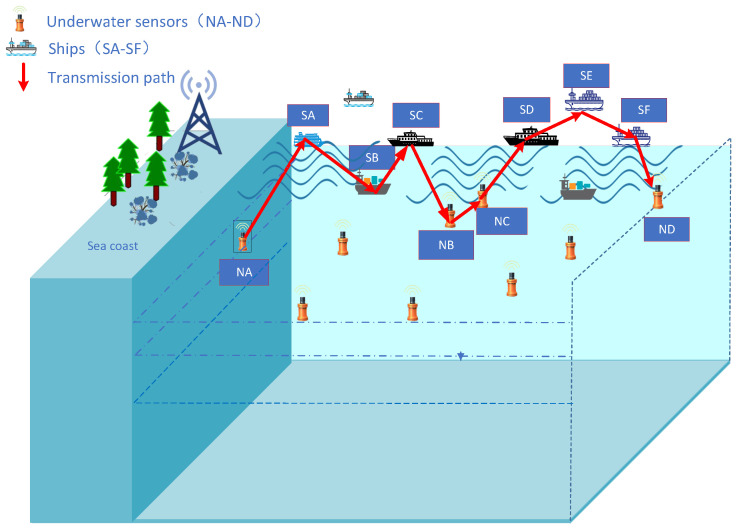
HDTA algorithm data transmission path.

As shown in Figure 2, when the underwater node NA has data to send to the underwater node ND, it selects the ship SA on the sea surface to forward the data. Then, ship SA forwards the data to ship SB, and ship SB forwards the data to ship SC. When ship SC cannot find any neighboring ships, it transmits the data to the underwater node NB. If NB cannot find any ships on the surface, it transmits the data to the underwater node NC. Node NC locates the ship SD on the surface and transmits the data. Ship SD forwards the data to ship SE, and finally, ship SD sends the data to the target node ND.

This study uses a Bayesian reputation-based spatial crowdsourcing allocation algorithm to select the next relay ship node. Since ships have stronger computational resources compared to underwater nodes and are more flexible, ships are prioritized as relay nodes. The Bayesian spatial crowdsourcing allocation algorithm not only considers the reputation value but also factors in the location of the target node and the occurrence of black holes. This ensures that the algorithm improves transmission efficiency while maintaining stable transmission and minimizes the length of the transmission path.

**Figure 3:**
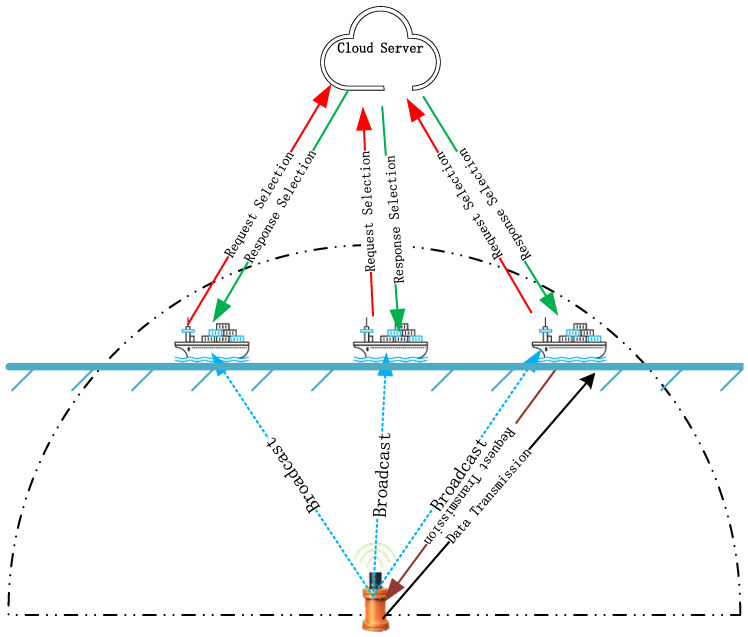
Information transmission from underwater node to ship.

Ships are capable of satellite communication, allowing direct communication with cloud servers via satellites. However, due to the high cost of satellite communication, it is not suitable for large-scale data transmission over long distances and is only used for path planning. During data transmission, data are transmitted via multi-hop communication between ships and nodes using wireless and acoustic communication methods. As shown in Figure 3 (the blue dashed line represents broadcasting, the red line represents request selection, the green line represents response selection, and the black line represents data transmission), when an underwater node collects data to send to other underwater nodes or to the shore station, it first sets a timer T and broadcasts to the ships within its communication range. If multiple ships respond, the responding ships will forward their information along with the pre-transmission information to the cloud server. The server uses the HDTA algorithm to calculate and update the ship’s reputation, selecting the ship with the highest overall score, which is the ship that the underwater node should select. The server then returns the selected ship’s information in an encrypted form to the ship, which then forwards it back to the underwater node. After decryption, the underwater node sends the data to the selected ship, completing the first phase of data transmission. When the timer T expires and no ship has responded, the node will send the data to the nearest underwater node, which will redistribute the task.

**Figure 4:**
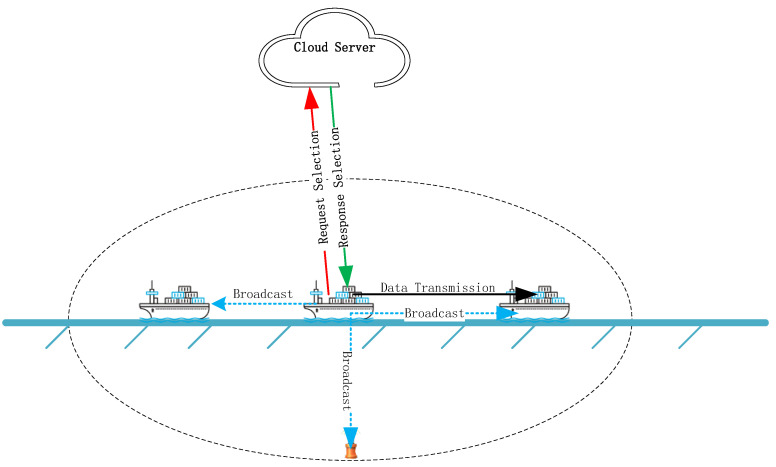
Information transmission from ship to other ships.

As shown in Figure 4, when a ship needs to transmit collected data to other ships, shore stations, or underwater nodes, it sets a timer T and broadcasts a data transmission request. If multiple units respond, the ship collects the positions of the responding ships and shore stations, then sends this information along with its own data back to the cloud server. After the cloud server processes the information using an algorithm, it returns the results to the ship. The ship that is sending the data uses the server’s selection to determine the next relay node to complete the data transmission.

If no ships or shore stations respond, the ship will wait for a period of time before sending another broadcast request. If the timer T expires and there are still no responses, the ship will send the data to the nearest underwater node, which will redistribute the task. The underwater node will issue the task, and if no ship accepts the task within a specified time D, it will send the data to the highest-priority underwater neighbor (i.e., the closest underwater node to the target node). This neighboring node will then initiate another task allocation process, continuing until a ship accepts the task. The data transmission path is established step by step, creating a complete transmission link from the source node to the destination node.

### 3.2. Spatial Crowdsourcing Task Allocation

#### 3.2.1. Basic Idea

The ship network consists of numerous ships. To effectively select the appropriate ships for establishing data transmission paths, this study adopts a spatial crowdsourcing approach. In this method, the underwater node acts as the task publisher, broadcasting task information that includes its own location, the target node’s location, and the time required for data transmission. And ships within the node’s communication range act as workers, receiving the information and deciding whether to participate in the crowdsourcing task. Then, participating ships (workers) will be rewarded. Ships determine their participation based on their navigation trajectory and speed. Ships that are about to leave the communication range of the node or those that will leave the area before completing the data transmission do not participate in the crowdsourcing task.

When the underwater node takes on the role of the task publisher, it broadcasts task information to potential ship workers within its communication range. These workers must meet specific threshold conditions. The algorithm sets a minimum threshold to filter out ships that do not meet the basic parameters, reducing the computational load. Only ships that meet the threshold criteria are considered eligible candidates to be processed further in the ship worker selection algorithm.

Ship workers that meet the threshold conditions use a Bayesian method to calculate the direct reputation value of the ship workers. They then combine this with the number of user participations to compute the ship’s total reputation value. A comprehensive scoring function is designed, taking into account factors like the target node’s location, ship density, and potential black holes that may affect the communication outcomes. The ship worker with the highest score according to this function is selected. The ship worker then evaluates whether to participate in the crowdsourcing task based on the task’s target location and corresponding reward. If the worker agrees to participate, the underwater node transmits the crowdsourcing information to the ship worker. If the worker rejects the task, the algorithm will continue to select candidate workers until a willing worker is found. Workers who reject tasks will not be included in the reputation evaluation process. On the other hand, once a worker accepts the task, their reputation is calculated. Upon task completion, the worker’s reputation will increase, while failing to complete the task will lead to a decrease in reputation. If a worker’s reputation falls below the minimum required reputation for the crowdsourcing task, they will no longer be allowed to participate in future tasks. The pseudo-code of the algorithm is presented in Algorithm 1.
**Algorithm 1:** Select ship for crowdsourcing task.1:**Input:** Task info (underwater node location, target node location, time cost), threshold values (minimum credibility, etc.)2:**Output:** Task transmitted or transmission failure3:function selectShipForCrowdsourcingTask()4:   taskInfo ← gatherTaskInfo()5:   ships ← scanForNearbyShips()6:   qualifiedShips ← filterShipsByThreshold(ships)7:**if** qualifiedShips is empty **then**8:    continueSelectAlgo()9:    continue10:**end if**11:selectedShip ← HDTAAlgorithm(qualifiedShips, taskInfo)12:**if** selectedShip is null **then**13:    continueSelectAlgo()14:    continue15:**end if**16:updateShipCredibility(selectedShip, taskInfo)17:sendTaskTo(selectedShip, taskInfo)18:**if** dataTransmittedToTarget(selectedShip, taskInfo) **then**19:    taskCompleted()20:**else**21:    transmissionFailed()22:**end if**

#### 3.2.2. Bayesian Reputation-Based Crowdsourcing Task Allocation Algorithm

A Bayesian-based reputation model aimed at evaluating the integrity level of workers is proposed. The model not only serves as an important metric for assessing worker quality but also plays a crucial role when workers participate in crowdsourcing tasks. During the crowdsourcing task allocation process, users with higher reputations should be given higher priority, while those with lower reputations should be given lower priority. And inspired by research in physical dynamics, which enhances robustness by adopting original methods for reputation allocation, this paper introduces an iterative reputation allocation process within the Rating-Based Preference Dynamics (RBPD) method. By utilizing an iterative reputation ranking based on the Beta probability distribution, we propose a Bayesian reputation-based spatial crowdsourcing allocation algorithm.

**Figure 5:**
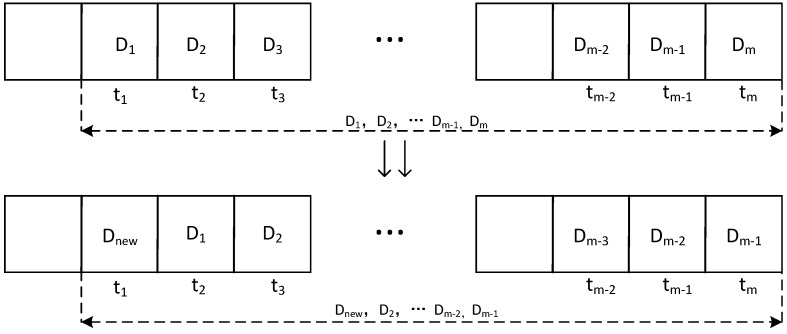
Time segment update process of reputation model.

In this paper, we use Bayesian methods to calculate the direct reputation values of eligible ship workers. First, the historical data of users are divided into *m* equal time periods, with each period representing a specific phase of user behavior. When calculating reputation values, different weights are assigned to each time period, with more recent periods receiving higher weights. In other words, the recent behavior of ship workers is given greater relative importance in the evaluation process. As shown in Figure 5, over time, the system automatically incorporates new data segments and gradually discards the oldest ones, ensuring that the evaluation results are updated in a timely manner. This approach ensures that the reputation values are continuously adapted to reflect the latest behaviors and performance of the workers.

The algorithm defines *m* as the total number of time periods, where $t_{1},t_{2},\ldots t_{m}$ represent each time period, with $t_{1}$ being the most recent time period and $t_{m}$ being the earliest time period. Different weights are assigned to each time period, and the direct weighted sum of credibility values is

(1) $$C=\sum_{t=1}^{m}w_{t}\cdot D_{t}$$

The term $w_{t}$ represents the weight corresponding to the time period *t*, and $D_{t}$ the credibility value for the time period *t*. When a new data segment $D_{new}$ is added, we have the following:

- The weight is updated to $w_{m}\leftarrow w_{m-1},w_{m-1}\leftarrow w_{m-2},\ldots ,w_{2}\leftarrow w_{1},w_{1}\leftarrow w_{new}$;
- The direct credibility value is updated to
  (2) $$C=w_{new}\cdot D_{new}+\sum_{t=1}^{m-1}w_{t+1}\cdot D_{t}$$

It uses Bayes’ formula to calculate the credibility value for each time segment of the ship. In the Bayesian framework, the initial assessment of user credibility (i.e., prior probability) is combined with the observed behavioral data (i.e., likelihood) to form an updated estimate of user credibility (i.e., posterior probability). The key advantage of this method lies in its natural ability to integrate new information, allowing the credibility evaluation to flexibly reflect the latest user behavior. The Bayes formula is

(3) $$P\left(H|D\right)=\frac{P(D|H)\times P(H)}{P(D)}$$

In this context, P(H|D) represents the posterior probability of assuming *H* to be true for given data *D*. P(H|D) represents the likelihood that *D* is observed if P(H) is true. P(H) represents the prior probability that hypothesis *H* is true. P(D) represents the total probability of observing data *D*.

To facilitate the expression and update of reputation, we use the Beta distribution as the prior probability distribution for the direct credibility value. The Beta distribution is preferred for modeling user reputation, and this model is used to calculate the user’s instantaneous reputation. A normalization method is applied to extract scores as positive or negative behaviors. By combining the current behavioral performance with the initial user reputation, the current instantaneous reputation is calculated. The probability density function of the Beta distribution can be expressed using the Gamma function as

(4) $$P\left(x;\alpha ,\beta \right)=\frac{x^{\alpha -1}{\left(1-x\right)}^{\beta -1}}{\frac{\Gamma (\alpha )\Gamma (\beta )}{\Gamma (\alpha +\beta )}}$$

where 0<x<1, α>0, β>0.

When the reputation system is initialized without other prior information, the study uses a uniform distribution as the initial prior setting for the reputation system. This choice is based on its non-informative nature. Suppose that the underwater sensor node interacts with the ship r+s times, where *r* represents the number of successful tasks completed by the ship, and *s* represents the number of tasks not completed. *x* represents the next event. When the shape parameters α=1 and β=1, the distribution becomes a uniform distribution. The prior reputation can be represented as Beta(1,1) in the model. Then, the posterior distribution of *x* can be expressed as

(5) $$P\left(x\right)=\frac{Bin(r+s,r)Beta(1,1)}{\alpha +\beta +1}=Beta\left(r+1,s+1\right)$$

From the formula, it can be seen that the posterior distribution of *x* is still a Beta distribution. Therefore, the reputation $R_{i}$ of ship user *i* in this time segment can be expressed as

(6) $$R_{i}=Beta\left(a_{i}+1,b_{i}+1\right)$$

$a_{i}$ represents the total number of successful tasks completed by ship *i* within the time segment, and $b_{i}$ represents the total number of tasks not completed by ship *i* within the time segment. The credibility value $R_{i}$ is inherently a probability function and recognizes its non-direct observability as a physical quantity. Therefore, a new method for quantifying credibility is proposed. The credibility metric $D_{i}$ defined by the algorithm provides a quantitative evaluation of credibility, which is calculated based on the expected value of $R_{i}$. Through mathematical derivation, the expression for the credibility metric $D_{i}$ is given by

(7) $$D_{i}=E\left[Beta\left(a_{i}+1,b_{i}+1\right)\right]=\frac{a_{i}+1}{a_{i}+b_{i}+2}$$

After assigning the corresponding weights to the credibility values of the historical time segments, the direct credibility value $C_{i}$ of ship *i* is given by the formula

(8) $$C_{i}=\sum_{j=1}^{n}w_{ji}D_{ji}$$

where $W_{ji}$ represents the credibility value weight of ship *i* in time segment *j*, and $D_{ji}$ represents the credibility value calculated in time segment *j*. The weight calculation formula $W_{ji}$ is as follows:

(9) $$W_{ji}=\frac{\beta \cdot e^{-\alpha \cdot t_{j}}}{\sum_{k=1}^{n}e^{-\alpha \cdot t_{k}}}$$

Here, the exponential decay $e^{-\alpha \cdot t_{j}}$ ensures that as time $t_{j}$ increases, the weight gradually decreases, with α representing the time decay coefficient. If α is large, the weight decays more quickly, meaning that more recent data will have a greater weight. β can be used to adjust the importance of the credibility score for a particular time segment.

#### 3.2.3. Cold Start Problem

When new users participate in crowdsourcing, there are no historical data, making it difficult for the system to accurately assess the credibility of the new users, which can lead to the cold start problem. This may affect the fairness and efficiency of task allocation. To avoid situations where new, high-quality ship workers are not allocated tasks due to the lack of historical credibility data, this study not only considers the direct credibility of the ship workers but also takes into account the amount of participation. New users or users with less participation will have an initial credibility score $N_{\circ }$. The total credibility $Q_{i}$ is calculated by combining the direct credibility value $C_{i}$ obtained from the above algorithm and the initial credibility value $N_{\circ }$ according to their respective weights. As the amount of participation increases, the weight of the initial credibility $N_{\circ }$ becomes smaller, while the weight of $Q_{i}$ becomes larger. Once the participation count (*n* > 5), the weight of the initial score $N_{\circ }$ becomes 0, and the total credibility value $Q_{i}$ is entirely determined by $C_{i}$. Therefore, the final credibility value of ship *i*, denoted as $Q_{i}$, is calculated as follows:

(10) $$Q_{i}=(1-\alpha {)C}_{i}+N_{o}\alpha$$

where $C_{i}$ is the direct credibility value of ship *i*, $N_{\circ }$ is the participation count score, and here, α is the weight coefficient. The formula for calculating the weight coefficient α is as follows:

(11) $$\alpha =\left\{\begin{array}{c} \frac{5-n}{10}\;0\leq n\leq 5\\ 0\;\;\;\;\;\;n>5 \end{array}\right.$$

#### 3.2.4. Comprehensive Evaluation

A comprehensive scoring function is defined based on the ship’s location, credibility value, and ship density. This function is designed to comprehensively evaluate the multiple characteristics of each candidate ship and calculate its overall suitability score. The score of each ship reflects its overall advantage as a relay node, with the ship having the highest score being selected as the optimal relay.

**Figure 6:**
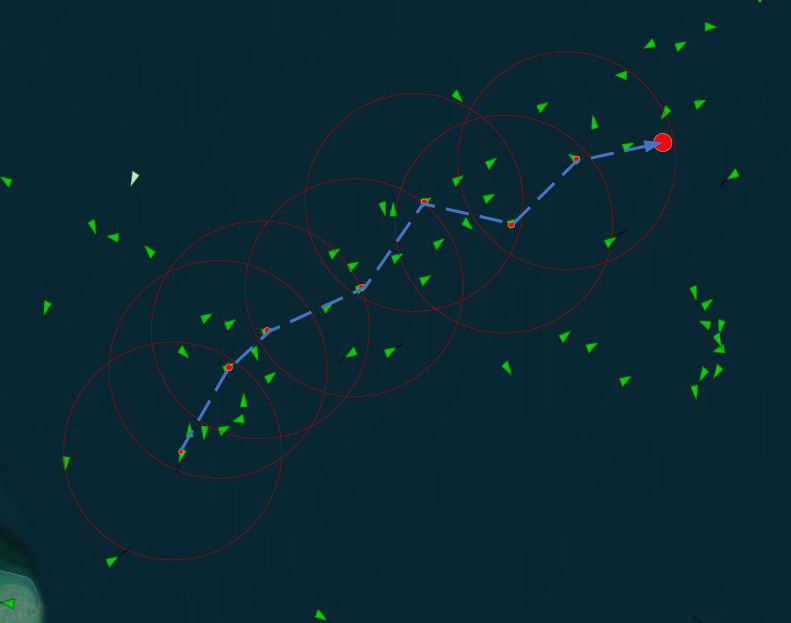
Transmission path. The green triangle represents ship nodes, the red dot represents the selected relay ship node, the red circle represents the transmission range, and the blue dashed arrow indicates the transmission direction.

Considering the impact of ship density around a node on communication effectiveness, communication transmission is more likely to be completed when the ship density is higher. The logistic function $DE_{i}=\frac{1}{1+e^{-k\cdot n}}$(n>0) is used as the ship density scoring function. $DE_{i}$ is the ship density score, *n* is the number of ships within the communication range of the node, and *k* is the adjustment parameter. When the number of surrounding ships is low, the density score approaches 1, while as the number of neighboring ships increases, the density score gradually increases. The synthetic scoring function is as follows:

(12) $$F\left(S_{i}\right)=\gamma \cdot Q_{i}+\delta \cdot DE_{i}+\eta \cdot D_{i}$$

where $F(S_{i})$ is the comprehensive score of the ship, while $Q_{i}$ and $D_{i}$ represent the credibility score and the distance score, respectively. γ, δ, and η are the weight coefficients for the corresponding factors. The comprehensive score function allows the algorithm to comprehensively evaluate the potential of each candidate ship as a relay node. It selects the ship with the highest comprehensive evaluation as the relay node to improve the overall communication network performance. The algorithm chooses the ship with the highest score as the final relay node. Then, the position coordinates of the information-carrying ship are updated, and the optimal relay ship for information transmission is selected, ensuring that the selected relay ship performs optimally in the current maritime communication environment. If a suitable relay ship is always available for selection, the relay selection strategy will quickly transmit the data to the target node. See Figure 6.

### 3.3. Incentive Mechanism

To stimulate the motivation and participation of ship workers in order to improve the quality of their work, this study adopts a platform-centered user incentive mechanism. Under this mechanism, ship workers receive certain material rewards after receiving task information and completing it. The results of the experimental study showed that the completion rate of tasks was significantly improved after the introduction of this mechanism. The amount of reward is mainly determined by five key factors: the size of the data packet, the number of task participation instances, the distance of transmission, the quality of work of the ship’s workers, and the density size of the ship. This dynamic compensation mechanism based on multiple factors aims to ensure the fairness and reasonableness of the compensation in order to motivate the ship workers to participate more actively and put in more effort to improve the task completion rate and work quality.

The calculation of the reward amount is mainly composed of the following components: the number of user participation component $Reward_{1}$, the user quality component (measured here by the user’s reputation value) $Reward_{2}$, and the ship density component of the region $Reward_{3}$. In addition, the size of the reward amount is directly and positively correlated with the size of the data packet and the distance of transmission.

In order to encourage new ship users to participate in the task, increase the participation of new users, and make them more willing to continue to participate in crowdsourcing tasks, ships that participate in the task for the first time are given an additional reward, that is, the initial value is greater, and with the increase in the number of tasks, the initial amount is returned to the normal level. This instant reward is a great way to encourage new ship workers to take on tasks. The formula of $Reward_{1}$ is as follows:

(13) $$Reward_{1}=\left[{\left(\frac{1}{2}\right)}^{n}+1\right]R_{e1}$$

In this formula, *n* is the number of times the user participates in any task, and $R_{e1}$ is the reward amount for $Reward_{1}$. The first participation by a new user can earn about twice the expected reward amount. As the number of participation instances increases, the reward grows, and $Reward_{1}$ approaches $R_{e1}$ in magnitude.

In order to improve the accuracy, completeness, and timeliness of information as well as to reduce errors and delays in information delivery, selecting high-quality workers is a good way to do this. High-quality users usually have higher reliability and can deliver information faster and better. The higher the quality of the ship’s workers, which means the higher the reputation value, the higher the amount of compensation will naturally be. The measure of worker quality remains the ratio of the current ship’s worker reputation value to the average of all ship’s workers’ reputation values. $Q_{p}$ and ${Reward}_{2}$ are calculated as follows:

(14) $$Q_{p}=\frac{Q_{i}}{Q_{avg}}$$

(15) $${Reward}_{2}=\frac{Q_{i}}{Q_{avg}}R_{e2}$$

where $Q_{i}$ is the current ship worker’s credibility value, $Q_{a\nu g}$ has the mean value of the ship worker’s credibility value, and $R_{e2}$ is the pre-amount of ${Reward}_{2}$.

For areas where the density of ship workers is sparse, it may lead to greater difficulty in information and data transmission. However, these areas are often important for data collection and play a key role in the accurate and timely transmission of information. When the density of ship workers in a region is relatively low, the challenge of insufficient personnel density can be compensated for by setting up extra rewards to remind ship workers that there are extra rewards in these regions, which can attract more ship workers from other regions to actively participate in the task. ${Reward}_{3}$ is calculated as follows:

(16) $${Reward}_{3}=\frac{\rho_{1}}{\rho_{i}}R_{e3}$$

where $\rho_{i}$ is the density of ships in the communication range where this ship or node is located, $\rho_{1}$ is the standard rated density, and $R_{e3}$ is the advance amount of $Reward_{3}$. So the payoff pay is calculated by the formula

(17) $$pay=\left(\left[{\left(\frac{1}{2}\right)}^{n}+1\right]R_{e2}+\frac{Q_{i}}{Q_{avg}}R_{e2}+\frac{\rho_{1}}{\rho_{i}}R_{e3}\right)D_{i}L_{i}$$

where $D_{i}$ is the size of the data volume of this task crowdsourced delivery data, and $L_{i}$ is the length of the Euclidean distance of this task crowdsourced delivery data.

## 4. Simulation Experiments

In this section of experiments, this study utilizes real ship data to evaluate the performance of the algorithm. The experimental platform is configured with Intel(R) Core(TM) i7-8750H CPU @ 2.1 GHz, 16.0 GB of RAM, and 64-bit Windows 11 as the operating system. The programming language is Python 3.10.7, and the development tool is PyCharm 3.10.7, while tools such as Matlab R2022b are used for data plotting and analysis.

**Figure 7:**
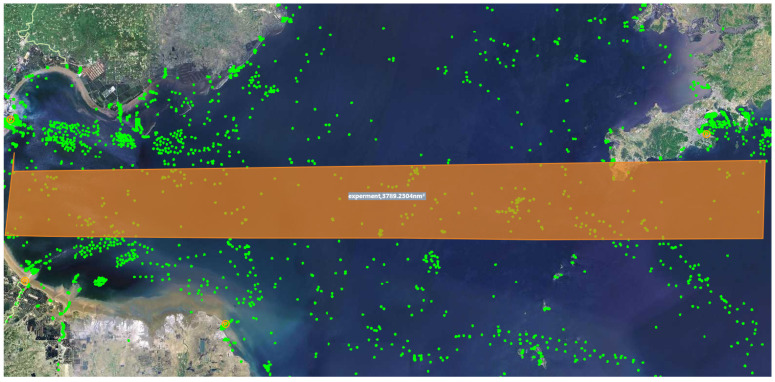
Ship real-time position map. The green dots represent the vessels, and the yellow area represents the experimental area.

**Figure 8:**
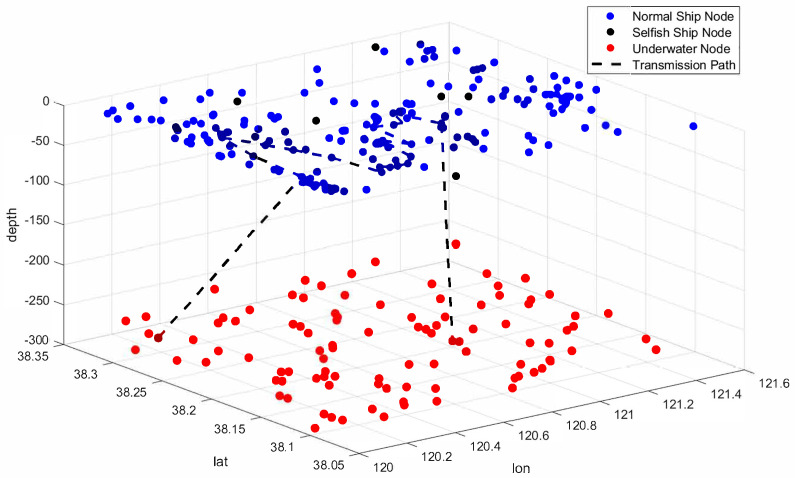
The 3D link transmission path.

In this study, real-time ship data provided by ShipShun (http://www.ships66.com) are used. Specifically, an area within the range of east longitude 38.461963 to 38.810483 and north latitude 119.066613 to 121.986084 at a certain moment on 8 January 2025 (shown in Figure 7) is selected, and the green points represent the real-time coordinate points of the ships. In this area, 738 ships are selected as real data for the experiment, with the assumption that each ship is equipped with a dual-head node, where each dual-head node is equipped with both above-water and underwater communication modules. In addition, the positions of 100 underwater sensor nodes are randomly generated within the range of east longitude 38.461963 to 38.810483, north latitude 119.066613 to 121.986084, and depth 0 to 300 m. The three-dimensional spatial distribution and transmission paths of the sensor nodes relative to the ship’s position are shown in Figure 8.

In order to simulate the real situation of real ship workers, ship workers are divided into two types: type a and type b. Type a ship workers have a high degree of trustworthiness, and their probability of successfully completing the task is set to be 98% in the experiment, which means that there is only a 2% probability of not being able to complete the task properly. Type b ship workers, on the other hand, belong to the selfish type, and their probability of not being able to complete the task is set to 90% in the experiment, and there is only a 10% probability of being able to complete the task. After sub-simulating the HDTA algorithm for 1000 times, it is found that an initial reputation value could be attached to each ship.

**Figure 9:**
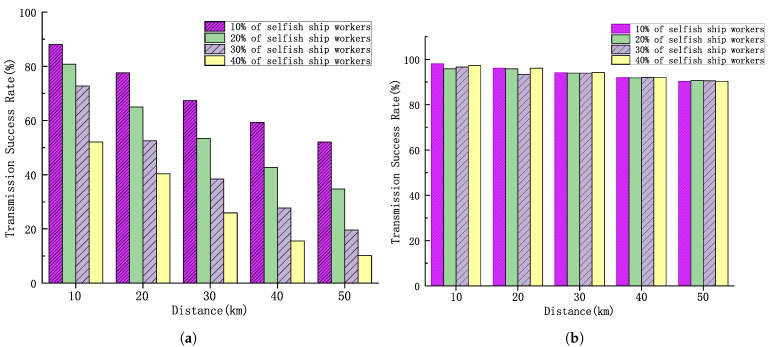
(**a**) Task success rate of the Greedy Forwarding algorithm versus transmission distance. (**b**) Task success rate of the HDTA algorithm versus the transmission distance.

As shown in Figure 9a, different percentages of selfish ship workers of 10%, 20%, 30% and 40% are set, and the success rate of task completion at different distances is analyzed. The results indicate that, within the same time frame, the task transmission success rate for the experimental group with a higher proportion of selfish ship workers is significantly lower than that for the experimental group with a lower proportion. Additionally, as the transmission distance increases, the task transmission success rates for experimental groups with different proportions of selfish ship workers all show a marked decline. This is because, as the transmission distance increases, the number of relay nodes encountered also increases, which in turn raises the likelihood of encountering selfish ship workers along the transmission path, ultimately leading to a decrease in the overall task transmission success rate.

Figure 9b illustrates the experimental results after the introduction of reputation selection. The changes in task completion rates at different distances are analyzed for 10%, 20%, 30%, and 40% selfish ship worker proportions. The comparison reveals that the task success rate stays at a high level for both increasing distance and increasing proportion of selfish ship workers. This is because the algorithm can efficiently filter out selfish ship workers and select the optimal ship workers to execute the crowdsourcing tasks.

**Figure 10:**
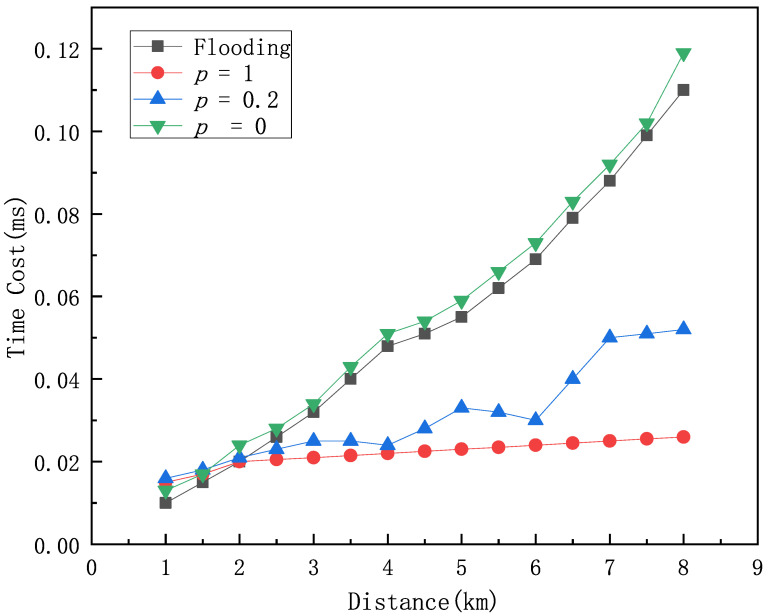
The impact of different vessel densities on the performance of the HDTA algorithm.

In order to investigate the impact of vessel density on data transmission speed, transmission reliability, and overall system performance, we simulate various vessel density scenarios. We analyze the effect of vessel density on the time overhead of the HDTA algorithm under conditions of the same maritime area, the same underwater node density, and the same proportion of selfish vessel nodes, and compare the results with those of the Flooding algorithm. The Flooding algorithm is a widely used network data transmission method that broadcasts data at each node in the absence of explicit routing information. Vessel density *p* represents the amount of vessels per square kilometer. We examine the time overhead for different vessel densities *p* = 1, *p* = 2, *p* = 3, and compare it with that of the Flooding algorithm as shown in Figure 10. The analysis shows that the ship density has a significant impact on the performance of the HDTA algorithm. When *p* = 0, it indicates that there are no ships in the surrounding area, and the HDTA algorithm relies entirely on the underwater network and underwater sensor nodes for multi-hop transmission. In this case, the algorithm performs at its lowest, with the highest time overhead. When *p* = 0.2, it represents a low-density ship area. The performance of the HDTA algorithm becomes unstable, as there may be no suitable ships available as relay nodes, and data can only be transmitted to nearby underwater sensor nodes or wait for a suitable ship. When the ship density reaches or exceeds *p* = 1, it is considered a high-density ship area, and the HDTA algorithm performs at its best.

**Figure 11:**
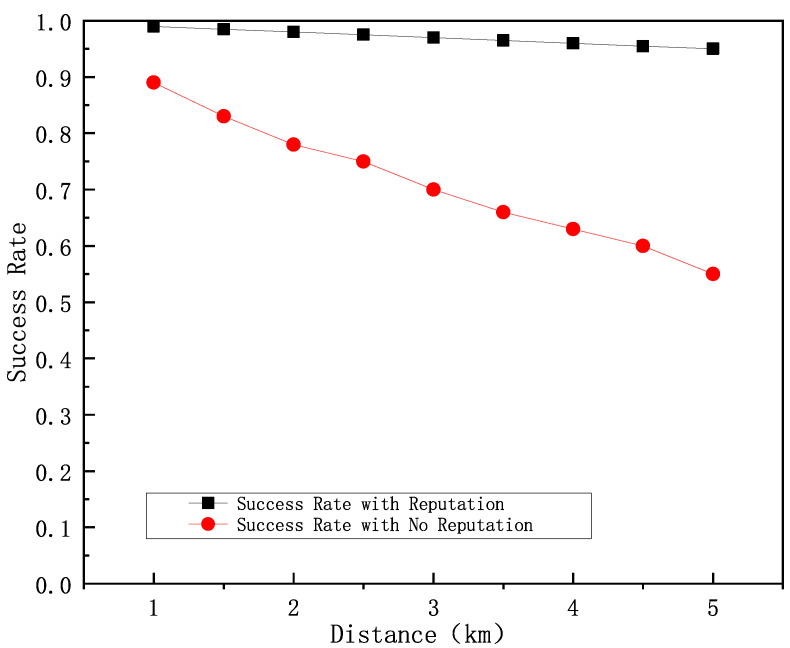
10% selfish ship worker task delivery success rate.

Figure 11 shows a comparison of the task transmission success rate of the algorithm with the addition of the reputation selection algorithm versus the algorithm without the addition of the reputation selection algorithm at different distances with 10% selfish ship workers. The results show that the task transmission success rate of the algorithm without adding reputation matching algorithm keeps decreasing as the distance increases, while the HDTA algorithm with the addition of the reputation selection algorithm always maintains a high and stable task transmission success rate.

**Figure 12:**
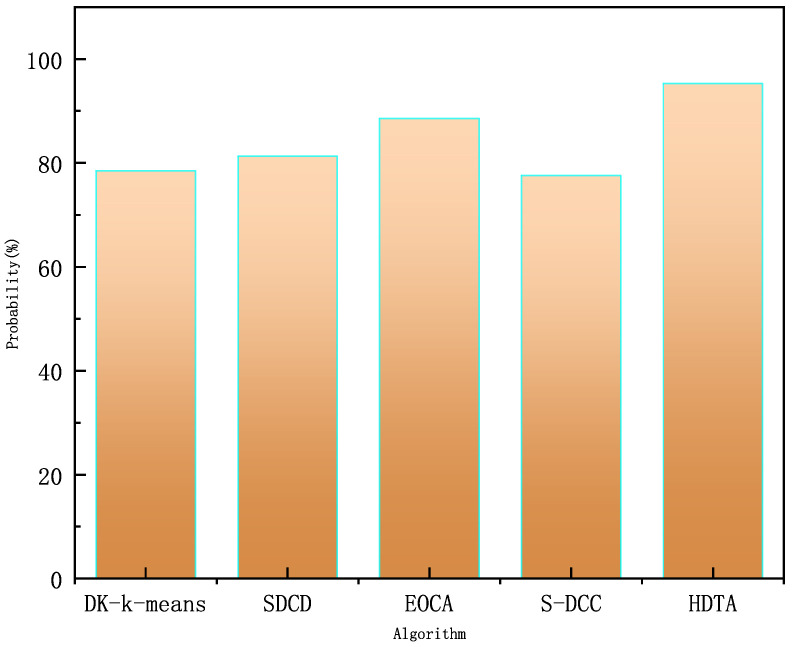
Packet transmission success rate.

Figure 12 illustrates the results of different algorithms in terms of packet transmission success rate. We compare advanced algorithms such as DC-K-means [25] for clustering algorithms to optimize multi-hop data transmission in underwater acoustic sensor networks, data collection based on a hierarchical approach for optimizing data transmission and energy management in underwater acoustic sensor networks. SDCS [26], energy-optimized clustering (EOCA) for multi-hop underwater acoustic cooperative sensor networks [39], selective dynamic coded cooperative communication (S-DCC) for multi-hop underwater acoustic sensor network [27], and other advanced algorithms, respectively, are subjected to 1000 simulations, and the probabilities of each algorithm successfully completing the packet transmission are 78.5%, 81.3%, 88.6%, and 77.6%, respectively, whereas the HDTA algorithm achieves a success rate of 95.3%. Based on the 95% confidence interval analysis, the success rates of the other algorithms are [77.1%, 79.9%], [80.0%, 82.6%], [87.4%, 89.8%], and [76.2%, 79.0%], while the success rate confidence interval for the HDTA algorithm is [94.5%, 96.1%].

Traditional multi-hop data transmission has a relatively low success rate due to factors such as channel quality, propagation conditions (e.g., signal attenuation and multipath effects), node energy, and state. In contrast, the HDTA algorithm leverages vessels as multi-hop relay nodes, which significantly improves performance. As the underwater part of the transmission is shifted to above-water, where vessel energy consumption is negligible, HDTA avoids many of the inevitable drawbacks of traditional multi-hop algorithms. Furthermore, the HDTA algorithm filters out selfish vessels and focuses on the recent behavior of vessel nodes. When a vessel’s behavior deviates from normal, its reputation is rapidly reduced, ensuring stable link transmission. These results indicate that the HDTA algorithm proposed in this paper outperforms other advanced algorithms in terms of packet delivery rate.

In underwater sensor networks, algorithms with low time complexity can reduce computational and energy consumption, improve real-time performance and stability, lower the communication overhead, and optimize overall performance. Especially in large-scale and dynamic environments, they ensure efficient operation, extend network lifespan, and enhance system reliability. In Table 2, we summarize and compare the complexity of crowdsourcing allocation algorithms from other references, and the results show that the HDTA algorithm has a clear advantage in terms of complexity.

**Figure 13:**
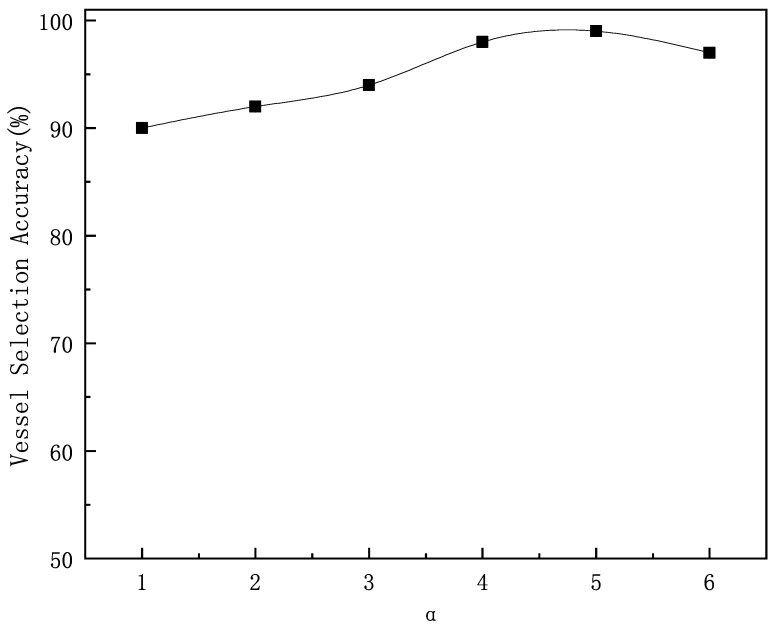
The effect of the time decay factor on algorithm performance.

As shown in Figure 13, the experiment analyzes the effect of the α time decay factor on the correctness of relay vessel selection. The experiment simulates 1000 selections of relay vessels under different α values. As α increases, the weight of more distant historical time slices decays faster, making the recent behavior have a greater impact on the vessel’s reputation value. This allows for a more accurate reflection of the vessel’s true reputation. When α = 4.5, the probability of selecting the correct vessel reaches its highest, with the algorithm’s success rate at 99.1%. Based on the 95% confidence interval analysis, the confidence interval for the algorithm’s success rate is [0.9851, 0.9969].

**Figure 14:**
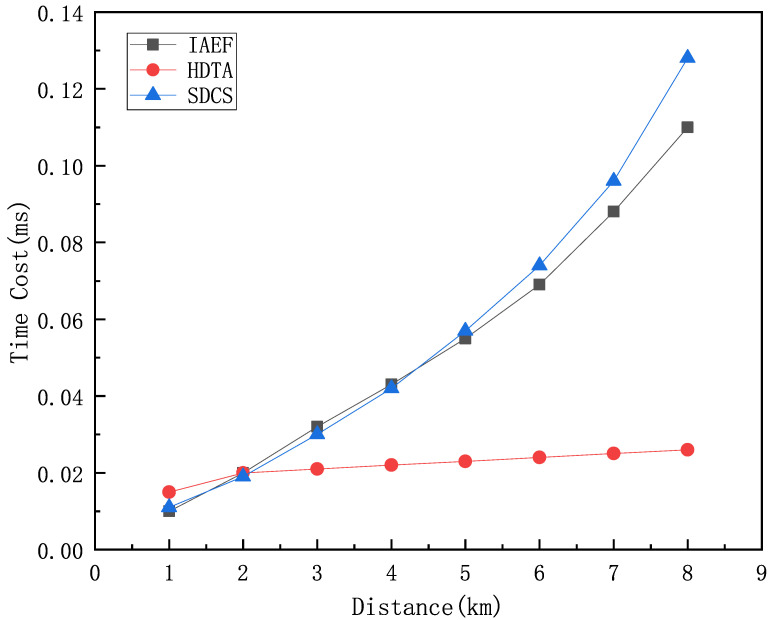
Comparison of the time overhead.

Figure 14 compares the time overhead of the HDTA algorithm with the IAEF algorithm [44] and SDCS [26] under the same packet size, transmission protocol, and transmission distance conditions. In the underwater part, the time overhead differences between the three algorithms are relatively small. When the target node is closer, the transmission path of the HDTA algorithm is mostly underwater, and the path length is slightly longer than that of the traditional multi-hop algorithm, resulting in a slightly higher time overhead. As the propagation medium and transmission bandwidth change, the propagation speed shows significant differences. With increasing the transmission distance, the time overhead of IAEF and SDCS increases significantly, while the HDTA algorithm, by selecting vessels as relay nodes and using Very High Frequency (VHF) radio for vessel-to-vessel communication, significantly increases the transmission rate, thus greatly reducing the time overhead.

**Figure 15:**
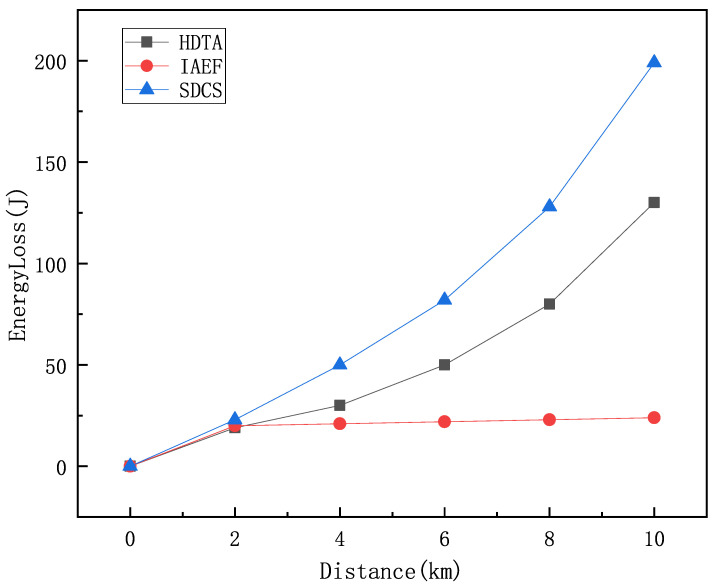
Comparison of energy loss.

Figure 15 compares the energy consumption of the HDTA algorithm with the IAEF and SDCS algorithms at different transmission distances. It can be seen that as the transmission distance increases, the energy consumption of the HDTA algorithm is significantly lower than that of IAEF and SDCS. This is because in the HDTA algorithm, the energy consumption of the vessels selected as relay nodes is negligible, while in traditional multi-hop algorithms, the energy consumption increases with distance. The HDTA algorithm reduces unnecessary energy consumption and increases the survival time of underwater nodes.

Dual-head nodes are relatively inexpensive to design and manufacture and are suitable for large-scale deployment on a wide range of vessels, including cruise ships, cargo ships, and fishing boats. Compared to the deployment of Unmanned Aerial Vehicle (UAV), Automated Underwater Vehicle (AUV), or Unmanned Surface Vehicle (USV) assisted data transmission solutions, dual-head nodes offer significant advantages in terms of cost and deployment efficiency. Additionally, dual-head nodes are relatively simple to maintain and upgrade, reducing the complexity and cost of long-term operations. By integrating with Automatic Identification Systems (AISs), dual-head nodes enable real-time transmission and sharing of vessel position and status information, thereby improving the efficiency and safety of maritime communications. Despite the technical feasibility and superiority of the HDTA algorithm, encouraging more ships to actively participate in crowdsourcing tasks remains a challenge in practical applications. Increasing vessel acceptance of HDTA and ensuring data privacy and security are key to promoting the technology.

## 5. Conclusions and Future Work

This paper proposes a dual-link data transmission method based on the Internet of Vessels, which significantly improves the reliability and transmission speed of underwater communication by utilizing vessels as relay nodes. The proposed spatial crowdsourcing task allocation algorithm, combined with a comprehensive evaluation function, selects the optimal relay nodes. The HDTA method demonstrates clear advantages in reducing transmission delay, increasing throughput, and improving network reliability, with simulation results validating its superiority. Future research will focus on the following aspects: further optimization of algorithm performance and computational efficiency, and strengthening the system’s robustness in complex marine environments, particularly in adapting to dynamic conditions such as weather changes and vessel density variations. Additionally, experimental validation in more real-world scenarios will be conducted, along with the development of new energy consumption models and energy-saving strategies, the integration of satellite communication and UAV-assisted communication technologies, and the realization of a land–sea–air integrated communication system. These efforts will ultimately provide more reliable and efficient solutions for data transmission in the marine domain in the future.

## Figures and Tables

**Table 1:** Comparison between our work and the related references.

References	Deep-Sea Advantages	Transmission Bandwidth	Stability	Deployment Cost	Energy Consumption	Long-Distance Transmission
[5,6]		✓	✓			
[8,9]		✓		✓		
[10]		✓			✓	
[25]	✓		✓	✓		
[26]	✓					
[27]		✓			✓	
HDTA		✓	✓	✓	✓	✓

**Table 2:** Time complexity of comparison algorithms.

Reference	Allocation Strategy	Analysis Model	Time Complexity
GREEDY [40]	Dynamic	AO	$O(n^{2})$
Allocation [41]	Static		$O(n^{2})$
LP-ALG [42]	Dynamic	KIID	O(n)
MMD-HST [43]	Dynamic	AO	$O(n^{2})$
HDTA	Static		O(n)

## Data Availability

The data presented in this study are available on request from the corresponding author.
